# Transplantation of rat cranial bone-derived mesenchymal stem cells promotes functional recovery in rats with spinal cord injury

**DOI:** 10.1038/s41598-021-01490-1

**Published:** 2021-11-09

**Authors:** Yuyo Maeda, Takashi Otsuka, Masaaki Takeda, Takahito Okazaki, Kiyoharu Shimizu, Masashi Kuwabara, Masahiro Hosogai, Louis Yuge, Takafumi Mitsuhara

**Affiliations:** 1 Department of Neurosurgery, Graduate School of Biomedical and Health Sciences, Hiroshima University, Kasumi 1-2-3, Minami-ku, Hiroshima, Japan; 2 Division of Bio-Environmental Adaptation Sciences, Graduate School of Biomedical and Health Sciences, Hiroshima University, Hiroshima, Japan

**Keywords:** Neurology, Medical research, Stem-cell research, Acute inflammation, Diseases, Trauma

## Abstract

Cell-based therapy using mesenchymal stem cells (MSCs) is a novel treatment strategy for spinal cord injury (SCI). MSCs can be isolated from various tissues, and their characteristics vary based on the source. However, reports demonstrating the effect of transplanted rat cranial bone-derived MSCs (rcMSCs) on rat SCI models are lacking. In this study, we determined the effect of transplanting rcMSCs in rat SCI models. MSCs were established from collected bone marrow and cranial bones. SCI rats were established using the weight-drop method and transplanted intravenously with MSCs at 24 h post SCI. The recovery of motor function and hindlimb electrophysiology was evaluated 4 weeks post transplantation. Electrophysiological recovery was evaluated by recording the transcranial electrical stimulation motor-evoked potentials. Tissue repair after SCI was assessed by calculating the cavity ratio. The expression of genes involved in the inflammatory response and cell death in the spinal cord tissue was assessed by real-time polymerase chain reaction. The transplantation of rcMSCs improved motor function and electrophysiology recovery, and reduced cavity ratio. The expression of proinflammatory cytokines was suppressed in the spinal cord tissues of the rats that received rcMSCs. These results demonstrate the efficacy of rcMSCs as cell-based therapy for SCI.

## Introduction

Spinal cord injury (SCI) can cause severe damage, leading to permanent loss of mobility, incontinence, and other functional losses. In the absence of effective treatments for SCI, surgical restabilization of the vertebral column and rehabilitation are currently the primary therapeutic options^1^. Cell-based therapy using mesenchymal stem cells (MSCs) has garnered attention as a novel approach for treating the damage caused by SCI. MSCs can be isolated from various tissues, such as bone marrow^2^ or adipose tissue^3^, and possess self-renewal and multilineage differentiation potentials. Some studies have revealed that the characteristics of MSCs may vary in these tissues^4–6^. In a previous study, researchers successfully established MSCs from rat and human cranial bones, and demonstrated that they secrete abundant neurotrophic factors when transplanted in a rat model of cerebral infarction^7,8^. However, reports demonstrating the effect of transplanted cranial bone-derived MSCs on rat SCI models are lacking. In addition, the mechanism of action of cranial bone-derived MSCs in central nervous system (CNS) disorders has not yet been investigated^7–9^. Motor function recovery has been evaluated only via behavioral assessments, as the electrophysiological recovery has not been determined. Furthermore, no studies have investigated the effect of transplanting MSCs on neurophysiological recovery over time using extended neurophysiological evaluation. We have previously established techniques for recording extended transcranial electrical stimulation motor-evoked potentials (tcMEPs) in rats^10^.

Therefore, in the present study, we investigated the effects of transplanting rat cranial bone-derived MSCs (rcMSCs) on the neurophysiology of an SCI rat model. Moreover, we determined the neurophysiological recovery using extended tcMEP recordings and the mechanism underlying rcMSCs function in an SCI model. We propose rcMSCs as a potential new source of cell therapy against SCIs.

## Results

### Flow cytometry analysis for MSC-specific markers

MSC-specific markers in rat bone marrow mesenchymal stem cells (rbMSCs) and rcMSCs were analyzed using flow cytometry. The results were positive for cell surface markers associated with MSCs, such as CD29, CD90, and CD44 (Supplementary Table S4 online); in contrast, they were negative for cell surface markers associated with hematopoietic cells, such as CD34 and CD45 (Supplementary Table S4 online). rbMSCs and rcMSCs exhibited similar characteristics, with their cell surface markers resembling those described in previous reports^7–9^.

### Multilineage cell differentiation

The differentiation potentials of the isolated rbMSCs and rcMSCs into osteogenic and adipocytic cells were investigated. Neither type of MSCs exhibited positive staining with Alizarin red S before osteogenic differentiation; however, positive cells were observed after differentiation (Supplementary Fig. S1A online), whereas staining with oil red O revealed that both types of MSCs tended to differentiate into adipocytic cells (Supplementary Fig. S1 online).

### Analysis of the genetic landscape of MSCs

**Figure 1:**
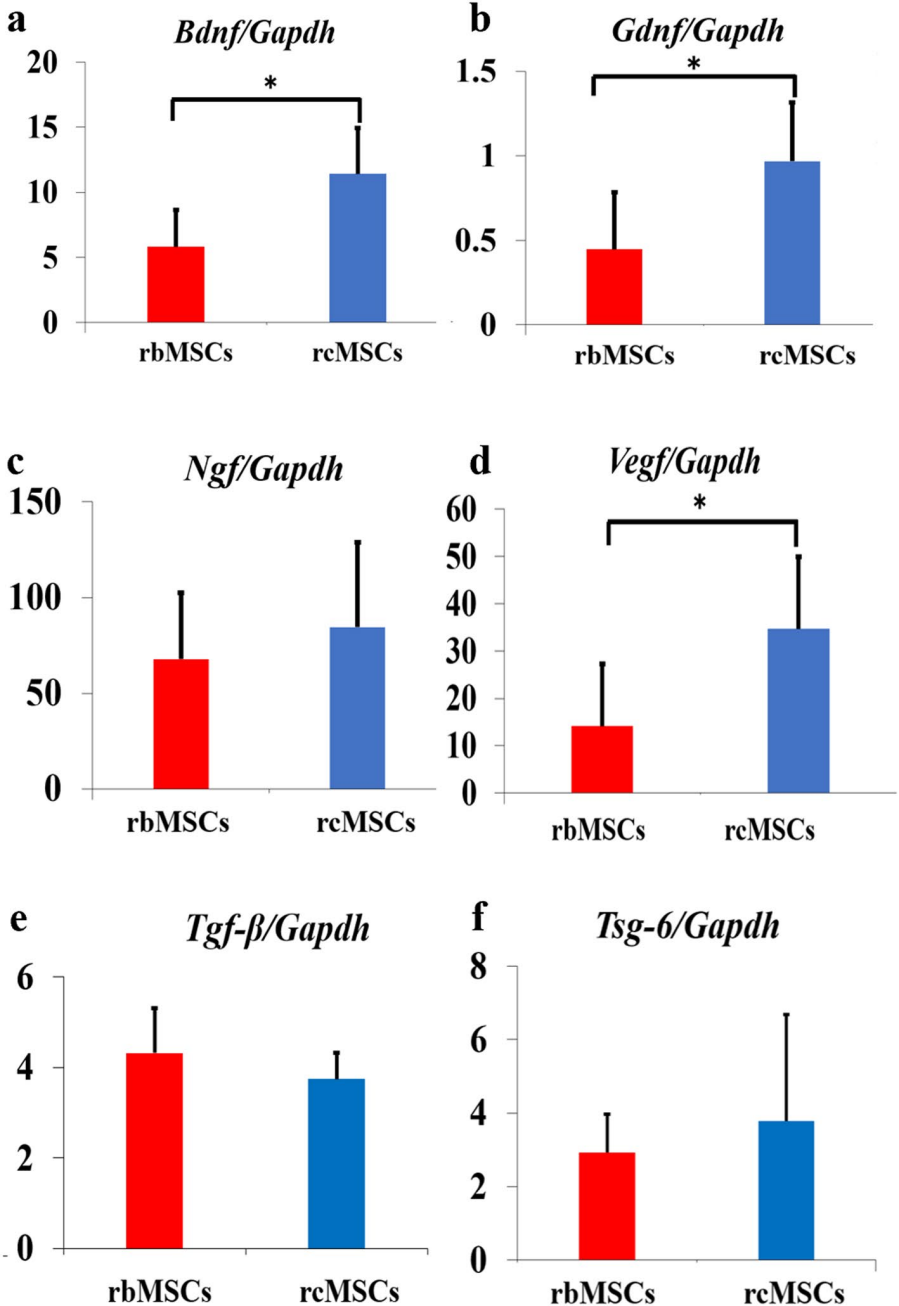
Real-time PCR analysis of neurotrophic and anti-inflammatory factor-associated genes. mRNA levels of *Bdnf* (**a**), *Gdnf* (**b**), *Ngf* (**c**), *Vegf* (**d**), *Tgfb* (**e**)*,* and *Tsg6* (**f**) relative to *Gapdh* expression. Data are presented as the mean ± SD of independent experiments (*P < 0.05, independent RNA extractions from biologically replicate cultures = 6).

The expression of genes encoding neurotrophic factors and anti-inflammatory factors in rbMSCs and rcMSCs was analyzed using real-time polymerase chain reaction (PCR). The expression of *Bdnf, Gdnf*, *Ngf*, and *Vegf,* which encode neurotrophic factors, and that of *Tgfb* and *Tsg6,* which encode anti-inflammatory factors, was evaluated. The expression of *Bdnf*, *Gdnf*, and *Vegf* was significantly higher in rcMSCs than in rbMSCs (*P* < 0.05, *P* < 0.05, *P* < 0.05, respectively; Fig. 1a,b,d). The expression of *Ngf* and of genes encoding anti-inflammatory factors did not differ significantly between rcMSCs and rbMSCs at passage 3 (Fig. 1c,e,f).

### Effects of cell transplantation in an SCI rat model: recovery of motor function

**Figure 2:**
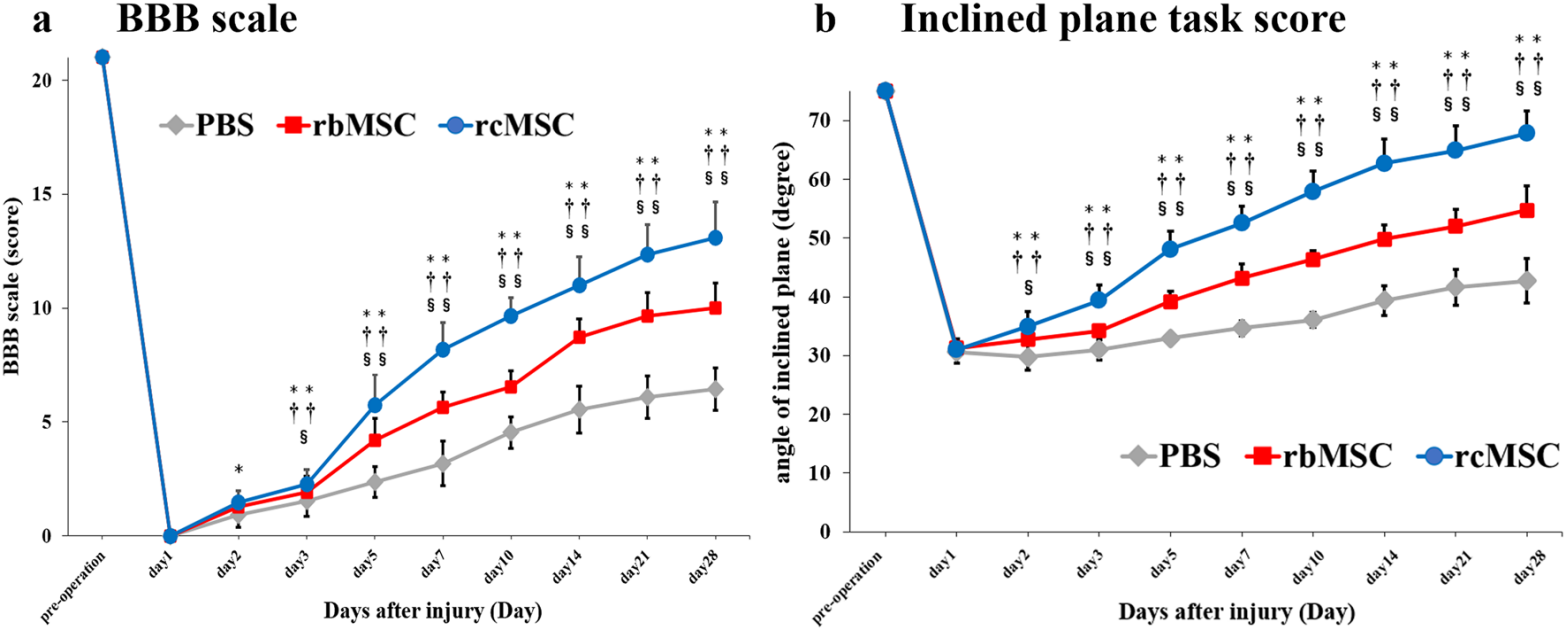
Effect of cell transplantation on motor function recovery. Results of the BBB scale (**a**) and inclined plane task score (**b**). Data are presented as the mean ± SD. * PBS group vs rcMSC group; ^†^PBS group versus rbMSC group; ^§^rcMSC group vs rbMSC group. Rats in the rcMSC group showed more significant improvements in BBB scale and inclined plane task score than rats in other groups (*P < 0.05, **P < 0.01, ^††^*P* < 0.01, ^§^*P* < 0.05, and ^§§^*P* < 0.01, respectively, n = 11).

We assessed the neurological function using the Basso–Beattie–Bresnahan (BBB) scale, and the inclined plane task score was used to assess the functional benefits after the transplantation of rbMSCs and rcMSCs post-SCI. All rats showed recovery of motor function in these tests after SCI. Improvements in the BBB scale score were significantly greater in the rcMSC group than those in the phosphate-buffered saline (PBS) group on day 1 (*P* < 0.05; Fig. 2a) and on days 3, 5, 7, 10, 14, 21, and 28 after injury (*P* < 0.01; Fig. 2a). In addition, improvements in the rcMSC group were also higher than those in the rbMSC groups on day 3 (*P* < 0.05; Fig. 2a) and on days 5, 7, 10, 14, 21, and 28 after injury (*P* < 0.01; Fig. 2a). Improvements in inclined plane task scores were significantly higher in the rcMSC group than in the PBS group at 2, 3, 5, 7, 10, 14, 21, and 28 days after injury (*P* < 0.01; Fig. 2b). In addition, improvements in the rcMSC group were also higher than those in the rbMSC group on day 2 (*P* < 0.05; Fig. 2b) and on days 3, 5, 7, 10, 14, 21, and 28 after injury (*P* < 0.01; Fig. 2b). Rats in the rcMSC group exhibited more motor function recovery than those in the other groups.

### Effects of cell transplantation in an SCI rat model: electrophysiological recovery

**Figure 3:**
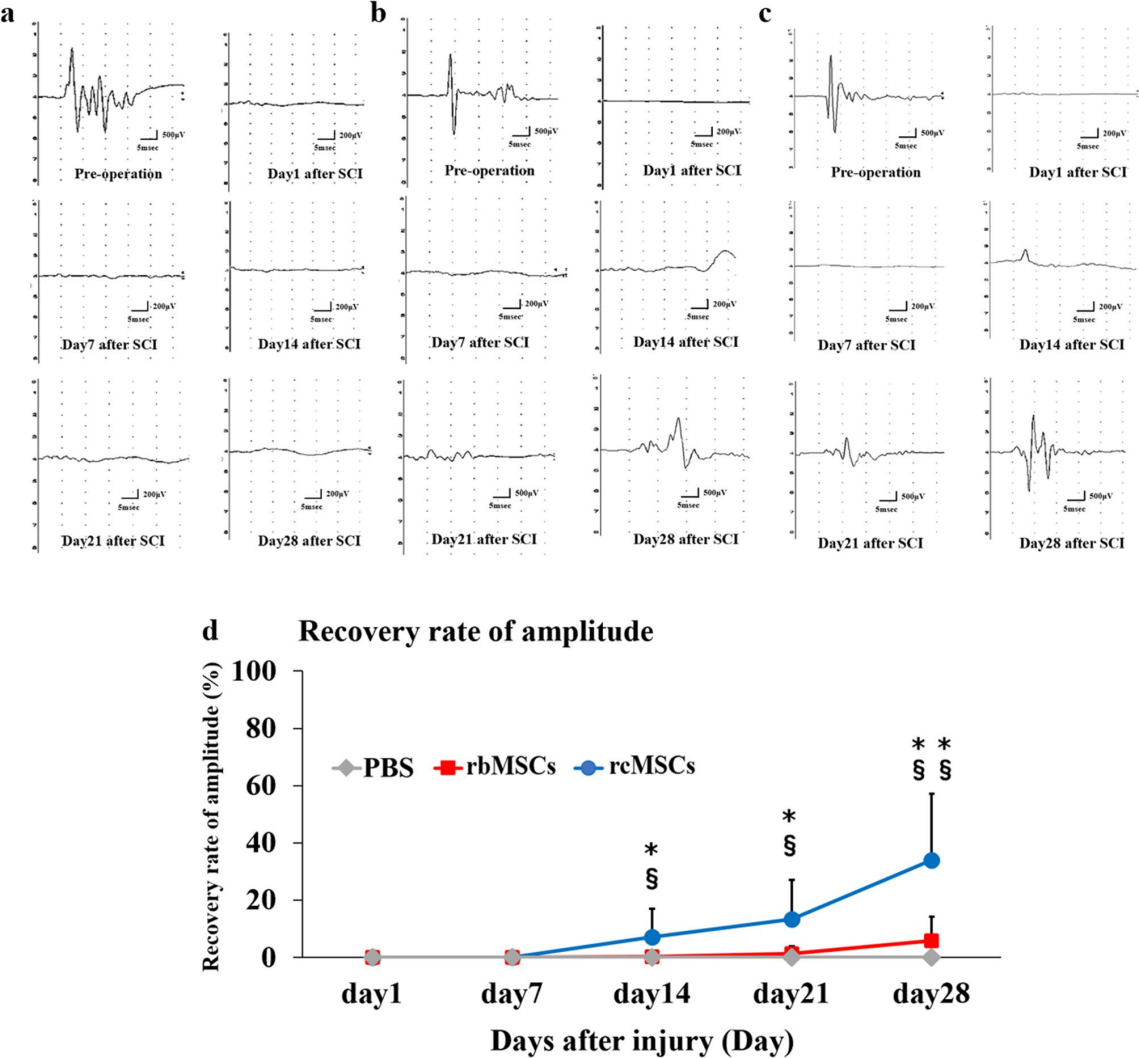
Effect of cell transplantation on electrophysiological recovery. Representative tcMEP recordings in the hindlimbs plotted at pre-operation, and at 1, 7, 14, 21, and 28 days after SCI (**a**: PBS group, **b**: rbMSC group, **c**: rcMSC group). Recovery rate of amplitude (**d**). Data are presented as means ± SD. *PBS group vs. rcMSC group; ^§^ rcMSC group versus rbMSC group. Rats in the rcMSC group showed more significant improvements in recovery rate of amplitude than those in other groups (*P < 0.05, **P < 0.01, ^§^*P* < 0.05, and ^§§^*P* < 0.01, respectively, n = 11).

Representative waveforms of tcMEPs pre-operation and at 1, 7, 21, and 28 days after SCI are shown in Fig. 3a–c. The waveform of tcMEPs disappeared and gradually recovered after SCI. The recovery rate of the amplitude improved at 14, 21, and 28 days after SCI compared with that on the day after injury in the rbMSC and rcMSC groups (Fig. 3d). In addition, the recovery rate of the amplitudes in the rcMSC group was higher than that in other groups on days 14 and 21 (*P* < 0.05; Fig. 3d) and at 28 days after SCI (*P* < 0.01; Fig. 3d).

### Effects of cell transplantation in an SCI rat model: cavity repair

**Figure 4:**
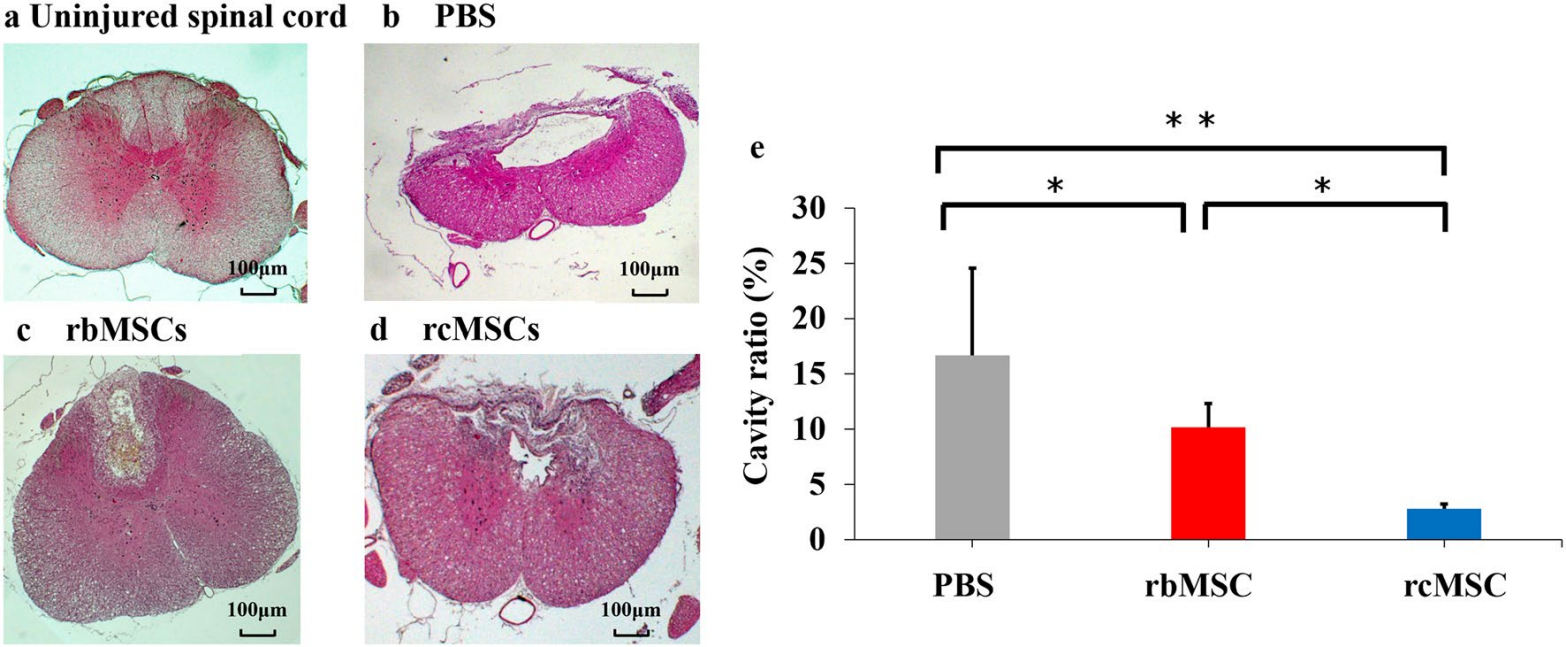
Cavity formation after spinal cord injury (SCI). Staining of the cavity formed after SCI (with hematoxylin and eosin). (**a**) Uninjured spinal cord, (**b**) PBS group, (**c**) rbMSC group, and (**d**) rcMSC group. The graph in (**e**) shows the difference in the volume of cavities at 28 days after SCI between the three groups. Data are presented as the mean ± SD. The cavity ratio in the rcMSC group was significantly lower than that in the three groups (*P < 0.05 and **P < 0.01, respectively, n = PBS: 6, rbMSC: 6, rcMSC: 5).

We assessed cavity repair using the cavity ratio and compared the histological recovery after the transplantation of rbMSCs and rcMSCs following SCI. Only small cavities were identified within the SCI lesions in the rcMSC group (Fig. 4a–d). The cavity ratios of the rbMSC and rcMSC groups were significantly lower than those of the PBS group (Fig. 4e). The cavity ratio of the rcMSC group was also significantly lower than that of the rbMSC group (*P* < 0.05; Fig. 4e).

### Spinal cord tissue sampling and mRNA expression analysis of the spinal cord lesion site

**Figure 5:**
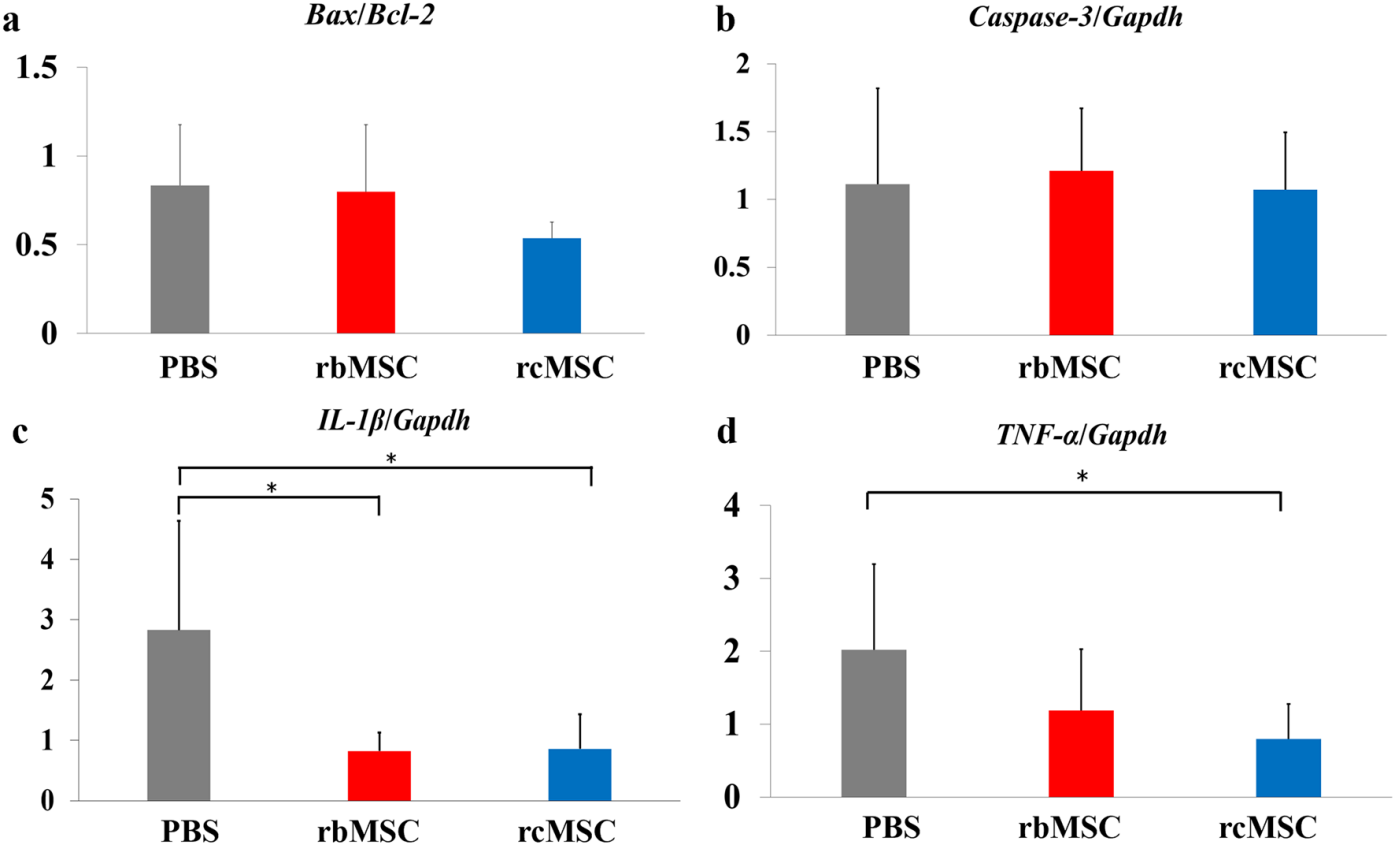
mRNA expression levels in the spinal cord lesion site. Relative mRNA levels of *Bax/Bcl2* (**a**), *Casp3/Gapdh* (**b**), *Il1B/Gapdh* (**c**), and *Tnfa/Gapdh* (**d**). Data are presented as the mean ± SD of independent experiments (*P < 0.05, n = PBS: 7, rbMSC: 6, rcMSC: 6).

The mRNA expression of *Bax/Bcl2* and *Casp3/Gapdh* did not differ significantly between the three groups (Fig. 5a,b). Moreover, the levels of *Il1b* were significantly lower in the rbMSC and rcMSC groups than in the PBS group (*P* < 0.05; Fig. 5c), and those of *Tnfa* were significantly lower in the rcMSC group than in the PBS group (*P* < 0.05; Fig. 5d).

### NG108-15 cell death assay after exposure to oxidative and inflammatory stress

**Figure 6:**
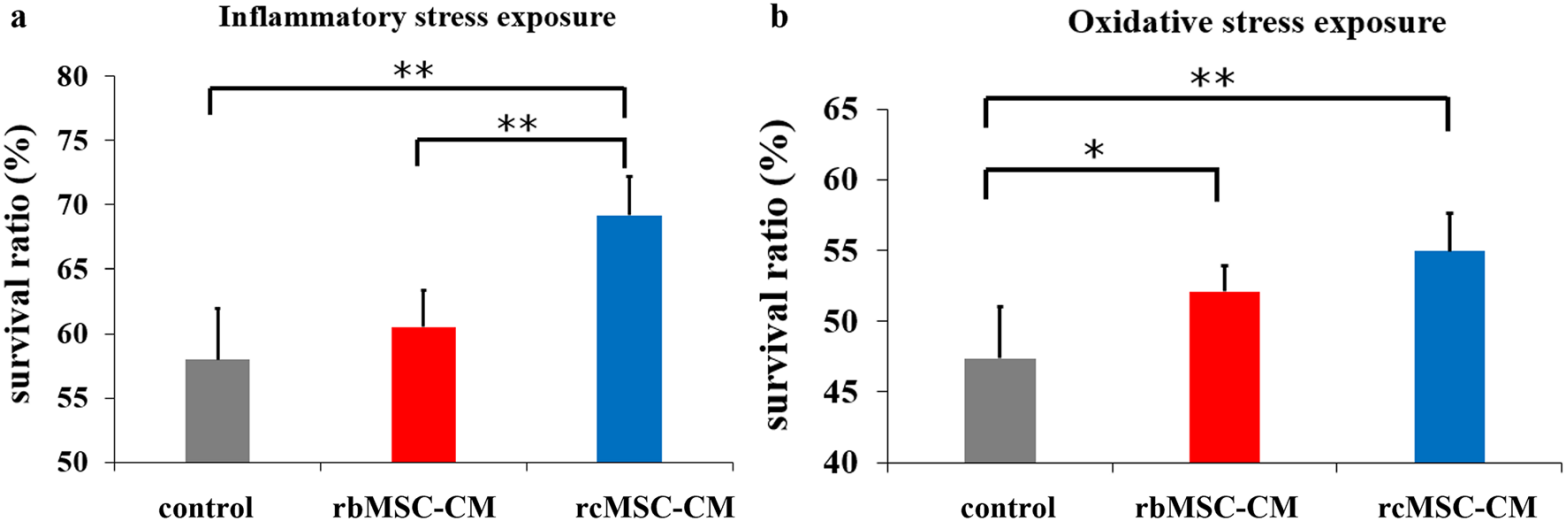
Effects of conditioned medium on the survival rate of stress-exposed NG108-15 cells. Survival rates of NG108-15 cells exposed to inflammatory stress (**a**) and oxidative stress (**b**). Data are presented as the mean ± SD of independent experiments (*P < 0.05 and **P < 0.01, respectively, n = 10).

The survival rate of NG108-15 cells exposed to inflammatory stress and cultured in rcMSC-conditioned medium (CM) was significantly higher than that in the other groups (*P* < 0.01; Fig. 6a). Moreover, the survival rate of NG108-15 cells exposed to oxidative stress and cultured in rcMSC-CM or rbMSC-CM was significantly higher than that in control cells (*P* < 0.01 and *P* < 0.05, respectively; Fig. 6b).

### Analysis of mRNA expression in stress-exposed NG108-15 cells

**Figure 7:**
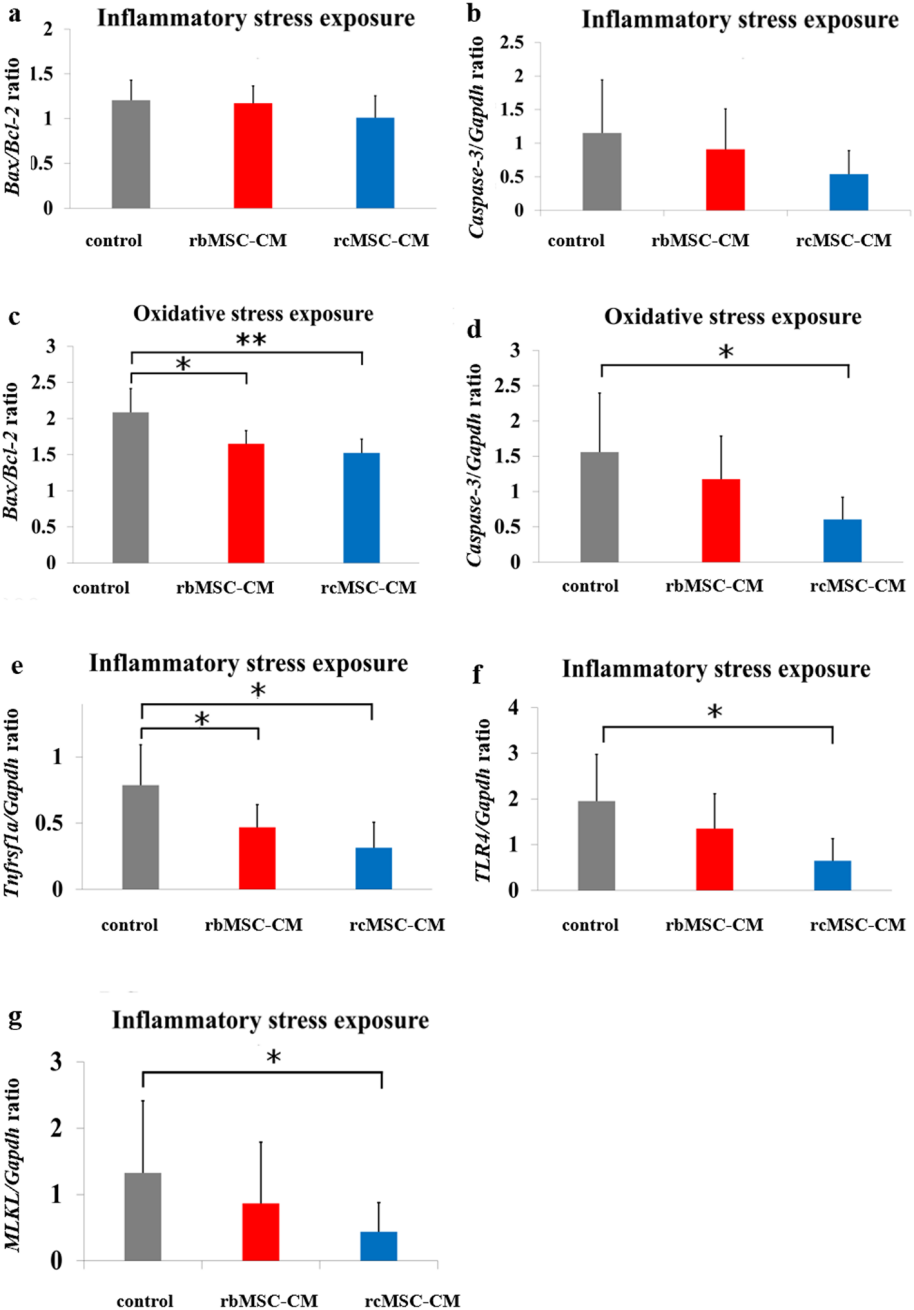
mRNA expression in stress-exposed NG108-15 cells. Relative mRNA levels of *Bax/Bcl2* (**a**) and*Casp3/Gapdh* (**b**) in inflammatory stress-exposed NG108-15 cells*.* mRNA levels of *Bax/Bcl2* (**c**) and *Casp3/Gapdh* (d) in oxidative stress-exposed NG108-15 cells. mRNA levels of *Tnfrs1a/Gapdh* (**e**), *Tlr4/Gapdh* (**f**), and *Mlkl/Gapdh* (**g**) in inflammatory stress-exposed NG108-15 cells*.* Data are presented as the mean ± SD of independent experiments (*P < 0.05 and ** *P* < 0.01, respectively, n = inflammatory stress-exposed: 9, oxidative stress-exposed: 10).

The *Bax/Bcl2* ratio and *Casp3* expression in inflammatory stress-exposed NG108-15 cells did not differ significantly between the three groups (Fig. 7a,b), whereas they were significantly lower in oxidative stress-exposed NG108-15 cells cultured in rcMSC-CM than in control cells (*P* < 0.05 and *P* < 0.01, respectively; Fig. 7c,d). The *Bax/Bcl2* ratio in cells cultured in rbMSC-CM was also significantly lower than that in control cells (*P* < 0.05; Fig. 7c). In addition, in inflammatory stress-exposed NG108-15 cells cultured in rcMSC-CM, the expression of *Tnfrs1a*, *Tlr4*, and *Mlkl* was significantly lower in control cells (*P* < 0.05; Fig. 7e–g). *Tnfrs1a* expression in cells cultured in rbMSC-CM was also significantly lower than that in control cells (*P* < 0.05; Fig. 7e).

## Discussion

Herein, we investigated the effectiveness of rcMSCs in SCI and aimed to prove the recovery of motor function electrophysiologically and clarify the detailed underlying mechanism. rcMSCs expressed high levels of neurotrophic factors and promoted motor function and electrophysiological recovery after transplantation in an SCI rat model, and their transplantation improved the neurological function more than that by the transplantation of rbMSCs. In the spinal cord lesion of the SCI, mRNA levels of *Il1b* and *Tnfa* were lower in the rcMSC group than in the PBS group. In vitro experiments further demonstrated that rcMSC-CM increased the survival rate of stress-exposed NG108-15 cells. The levels of *Tnfrs1a*, *Tlr4,* and *Mlkl* were significantly lower in the inflammatory stress-exposed NG108-15 cells cultured in rcMSC-CM than those in control cells. In addition, the *Bax/Bcl2* ratio and *Casp3* levels were significantly lower in oxidative stress-exposed NG108-15 cells cultured in rcMSC-CM than in control cells.

A previous study has shown that a part of the rat pyramidal tract runs on the posterior cord in the spinal cord^11^. Furthermore, several groups have used tcMEPs to study the motor pyramidal tract and neurological recovery in rat models of CNS injury^12–15^. Based on these previous findings, we concluded that tcMEPs can be used for behavioral evaluation and for the elucidation of the process of nerve function recovery in a rat model of posterior cord injury. Longitudinal evaluation of tcMEPs is also required for behavioral evaluation. However, previous studies on the effect of MSC transplantation have not evaluated tcMEPs longitudinally. In our study, we recorded tcMEPs in a time-dependent manner. Specifically, in the rcMSC group, the amplitude of tcMEPs gradually recovered and correlated with the recovery of motor function. These data suggested that rcMSCs transplantation resulted in the recovery of neural connections and regeneration of nerve fibers, playing a role in conduction^1^. The recording of tcMEPs in cases of severe motor paralysis is challenging^16,17^. Accordingly, the waveform of tcMEP could not be recorded in the present study when the BBB scale and degree of the inclined plane test score were low, such as in the acute phase after SCI. The appearance and improvement of the waveforms of tcMEP following motor function recovery are consistent with the observations of previous clinical reports^16,18^. Furthermore, as reported in humans, the existence of a measurable threshold for motor paralysis is also suspected in tcMEP recording of rats^16,18^.

Rats in the rcMSC group exhibited significantly higher functional recovery and a lower cavity ratio than those in the rbMSC and PBS groups. The levels of *Il1b* and *Tnfa* at the spinal cord lesion site of rats in the rcMSC group were significantly lower than those in the PBS group. Previous studies have shown that both IL-1β and TNF-α are upregulated after nerve injury^19,20^. In addition, the expressions of pro-inflammatory cytokines, such as TNF-α and IL-1β, increased after SCI^20,21^. These cytokines induce an inflammatory response, increase vascular permeability, elevate the levels of reactive oxygen species, and finally cause cell death via apoptosis and necroptosis^22–24^. TNF-α acts as a death receptor ligand in the necroptosis pathway^25^. Cell death via necrosis and necroptosis is involved in the formation of cavities in the injured spinal cord tissue^26^. The effect of transplanting MSCs derived from bone marrow and adipose tissue on these inflammatory cytokines has also been reported in SCI models^21,27,28^. In contrast, the effect of transplanting MSCs derived from cranial bone into an SCI model has not been reported. To the best of our knowledge, this is the first study to suggest that the transplantation of rcMSCs may be effective against inflammatory stress and cell death in injured tissue of the spinal cord by suppressing the expression of pro-inflammatory cytokines such as TNF-α and IL-1β.

The role of oxidative and inflammatory stress in the injured tissues of the spinal cord has been reported^29^. The results of our in vitro experiments using stress-exposed NG108-15 cells are in accordance with those obtained in the in vivo experiments. The survival rate of the NG108-15 cells was significantly higher in the rcMSC-CM group than that in the control group following exposure to oxidative or inflammatory stress. The *Bax/Bcl2* ratio and *Casp3* expression were significantly lower in the rcMSC-CM group of NG108-15 cells exposed to oxidative stress than in the control group. *Bax* is active upstream of the apoptotic cell death pathway, while caspase-3 is a downstream player^30,31^. Our results demonstrated that rcMSC-CM suppressed cell death under conditions of oxidative stress via the apoptotic pathways. *Tnfrs1a*, *Tlr4,* and *Mlkl* levels were significantly lower in the rcMSC-CM group of NG108-15 cells exposed to inflammatory stress than in the control group. TNFRS1A and TLR4 are membrane-bound receptors of TNF-α in the necroptosis pathway^32,33^. In addition, RIPK3, of the RIPK1-RIPK3-MLKL complex (complex IIb), perturbs the cell membrane by phosphorylating MLKL, leading to lysis and necroptosis^34^. Our results demonstrated that rcMSC-CM suppressed cell death under conditions of inflammatory stress via the necroptosis pathway.

The suppressive effect of neurotrophic factors such as GDNF^35,36^, BDNF^37,38^, VEGF^39^, and NGF^40^ on the apoptotic pathway has been reported. Similarly, studies have reported the suppression of the necroptosis pathway by GDNF, VEGF, and NGF owing to their anti-inflammatory properties^41,42^. We have previously reported that the expression of BDNF and NGF was significantly higher in rcMSCs than in rbMSCs^7,43^. In this study, the levels of GDNF, BDNF, and VEGF were significantly higher in rcMSCs than in rbMSCs. These results suggest that rcMSCs may exert a stronger anti-apoptotic and ant-necroptosis effect via the neurotrophic factors than rbMSCs. Reports focusing on the role of the necroptosis pathway in the context of MSC transplantation are limited. Our results suggest that the rcMSC-transplantation affects both the necroptosis pathway and the apoptosis pathway in SCI tissue. In addition, the high expression of neurotrophic factors in rcMSCs can be expected to modulate the effects of transplantation in CNS disorders.

This study has certain limitations. Although behavioral and electrophysiological evaluations, such as the analysis of tcMEPs, were conducted, the sensory function could not be evaluated. In future studies, we plan to evaluate sensory disorders based on electrophysiological evaluations, such as somatosensory evoked potential, and study the therapeutic effect of rcMSCs in models of CNS injury.

## Conclusions

We have provided novel evidence showing that cell-based therapy using rcMSCs results in excellent functional and electrophysiological recovery in an SCI rat model. We have also shown that rcMSCs exert anti-apoptotic and anti-necroptotic effects on the SCI tissue, suggesting that rcMSCs may be a new therapeutic agent in cell-based therapy for SCI.

## Methods

### Ethics statement

All methods have been reported in accordance with the ARRIVE guidelines (https://arriveguidelines.org) and the American Veterinary Medical Association (AVMA) Guidelines for the Euthanasia of Animals (2020). All study protocols were approved by the Animal Testing Committee Guidelines of Hiroshima University, Japan, and conducted under the authority of the Project License (A19-73).

### Isolation and culture of rbMSCs and rcMSCs

rbMSCs and rcMSCs were established as previously described^44^. For the isolation of MSCs, we collected the femur, tibia, and cranial bones from adult female Sprague–Dawley (SD) rats. Bone marrow and cranial bones were seeded on culture dishes. Once cells had adhered to the bottom of the culture dish, approximately 5–7 days, nonadherent cells were eliminated by changing the culture medium, and the adherent cells were used as rbMSCs and rcMSCs. The cells were maintained at 37 °C in an atmosphere of 5% carbon dioxide (CO_2_)/95% air, and the culture medium was changed every 3 days after detection of cell adhesion. The collected rbMSCs and rcMSCs were passaged at more than 80% confluence.

### Multilineage cell differentiation

rcMSCs and rbMSCs at passage 3 were used for differentiation into osteoblasts or adipocytes, as previously described^7,43^. Briefly, the cells were cultured in a mesenchymal stem cell osteogenic differentiation medium (Promocell, Heidelberg, Germany) and a mesenchymal stem cell adipogenic differentiation medium (Promocell) to induce osteogenic differentiation and adipogenic differentiation, respectively. Alizarin red S (Sigma-Aldrich, St. Louis, United States) and oil red O (Wako Pure Chemical Industries, Osaka, Japan) were used to stain the cells to confirm calcium deposition and lipid droplets, respectively.

### Flow cytometric analysis for MSC-specific markers

The analysis of MSC-specific markers was performed as previously described^7,43^. Confluent cultured rbMSCs and rcMSCs at passage 3 were collected using TrypLE™ Select (Thermo Fisher Scientific) and resuspended in PBS. Aliquots containing 1 × 10^5^ cells were incubated with phycoerythrin (PE)- or fluorescein isothiocyanate (FITC)-conjugated antibodies against rat CD45, CD90, and CD29^45^ (BD Biosciences, San Jose, CA), CD44 (BioLegend Co., San Diego, CA, USA), and CD34 (Santa Cruz Biotechnology, Dallas, TX, USA). PE-conjugated antibodies against CD29 and CD90 and FITC-conjugated antibodies against CD44 were used as MSC markers, and PE-conjugated antibodies against CD34 and FITC-conjugated antibodies against CD45 were used as hematopoietic markers. PE-conjugated mouse IgG1 and FITC-conjugated mouse IgG1 (BioLegend) were used as isotype controls. Data acquisition and analyses were performed three times for each type of MSC using FACS-Verse (BD Biosciences).

### Reverse transcription and real-time PCR

In earlier studies, we reported the gene expression profile of rcMSCs^7,43^. In the present study, we added neurotrophic factors and anti-inflammatory markers to further investigate gene expression. Total RNA was extracted from rbMSCs and rcMSCs at passage 3 according to the protocol accompanying the RNA extraction kit. Real-time PCR analyses were performed using a 7900 HT real-time PCR system (Applied Biosystems, Carlsbad, CA, USA). Here, we investigated the expression of the genes encoding for rat brain-derived neurotrophic factor (*Bdnf*), glial cell-derived neurotrophic factor (*Gdnf*), nerve growth factor (*Ngf*), and vascular endothelial growth factor (*Vegf*). In addition, we investigated the expression levels of the genes encoding the following anti-inflammatory markers: transforming growth factor-β (*Tgfb*) and tumor necrosis factor-stimulated gene-6 (*Tsg6*). The gene encoding glyceraldehyde-3-phosphate dehydrogenase (*Gapdh*) was used as the internal control to which the relative quantity of each mRNA was normalized. Primers used for TaqMan gene expression assays are listed in Supplementary Table S1.

### Animals and surgical procedure

Adult female SD rats (Charles River, Kanagawa, Japan), with a mean weight of 273 g (range, 250–300 g), were used for constructing an SCI model using the weight-dropping method^46,47^. The rats were anesthetized via the inhalation of 1.5% isoflurane. A midline linear incision was made over the thoracic (Th) 9–11 spinous processes. Laminectomy was performed at Th10. An impactor rod was set on the surface of the spinal cord at Th10 and a cylindrical brass weight (10 g) was dropped on the impactor. A spinal cord contusion was made with a force of 50 g/cm. Following contusion, the skin was sutured to close the lesion.

### Experimental groups and cell transplantation

Confluent cultured rbMSCs and rcMSCs at passage 3 were collected for transplantation. We divided the SCI rats into the following three groups according to the treatment received. The PBS group included rats receiving PBS only; the rbMSC group included rats transplanted with rbMSCs; and the rcMSC group included rats transplanted with rcMSCs. Eleven rats were prepared in each group. MSCs (1.0 × 10^6^ cells/300 µL PBS) were injected via the tail vein 24 h after SCI induction in the rbMSC and rcMSC groups. Considering the similar numbers of animals in published reports on rcMSCs^7,8^, we deemed our sample size sufficient to assess our model.

### Motor function recovery

The BBB scale^47–50^ and inclined plane task score^51^ were used for determining the behavioral endpoints of the motor function recovery after SCI. In this study, behavioral analysis was performed immediately before the spinal injury and every day from pre-operation to days 1, 2, 3, 5, 7, 10, 14, 21, and 28 after surgery. All motor functions were evaluated by an observer blinded to the group identities.

### Electrophysiological recovery

tcMEP was used as the neurophysiological endpoint of the motor electrophysiological recovery after SCI. Extended tcMEP was recorded using a bone-thinning technique^10^. To prepare for recording, the skulls of the rats were thinned with a diamond drill at the bregma and lambda positions. Two monopolar needle electrodes (Natus, Middleton, WI, USA) were used for stimulation. Transcranial electrical stimulation was performed with a train of four stimuli administered across a total duration of 0.5 ms using subdermal needle electrodes. The average value of the stimulation was 72 μV, and the tcMEP was recorded from the needle electrodes inserted in the quadriceps femoris of the rat hindlimb. The stimulation and recordings were performed using Endeavor CR (Nicolet Biomedical, Inc., Madison, WI, USA). tcMEPs were recorded pre-operatively and on days 1, 7, 14, 21, and 28 after surgery. Onset amplitudes were recorded, and the recovery ratio of the amplitudes was calculated by dividing the amplitude at each point by that at pre-operation.

### Histological analysis

We determined the cavity area ratio as an endpoint of histological recovery, as described in our previous paper^40–46^. Briefly, four weeks after transplantation, spinal cords were removed. The segments were mounted on microscope slides, and hematoxylin and eosin (H & E) staining was performed. H & E-stained segments were examined using a multifunctional microscope (BZ-9000; KEYENCE Co.). The cavity areas in injured rats were measured in each group using the digital image-processing software ImageJ (National Institutes of Health, Bethesda, MD, USA). The cavity ratio of each group was calculated by dividing the cavity area by the total coronally resected spinal cord area^46^.

### Spinal cord tissue sampling and analysis of mRNA expression at the site of the spinal cord lesion

The rats were anesthetized 24 h after MSC transplantation. Spinal cord tissues were removed and soaked in RNA Later (Sigma-Aldrich). Total RNA was extracted from the injured spinal cord segments (the length of the segments was 2 mm, centered on the lesion site). RNA extraction and reverse transcription were performed as described above. Real-time PCR was performed using oligonucleotide primer sets corresponding to the cDNA sequences of rat B-cell leukemia/lymphoma 2 protein (*Bcl2*), Bcl2-associated X protein (*Bax*), caspase-3 (*Casp3*), tumor necrosis factor-alpha (*Tnfa*), and interleukin-1 beta (*Il1b*). *Gapdh* was used as an internal control. The TaqMan gene expression assays used in this study are listed in Supplementary Table S2.

### Preparation of MSC-CM and NG108-15 cell cultures

MSC-CM was prepared as previously described^7,8^. Briefly, rbMSCs and rcMSCs were cultured, and the culture medium (rbMSC-CM and rcMSC-CM, respectively) was collected. In addition, the culture medium in which MSCs were not cultured was collected as control-CM. Neuroblastoma-glioma hybrid cells (NG108-15; ECACC, Porton Down, UK) were cultured in each of these media.

### NG108-15 cell death assay after exposure to oxidative and inflammatory stress

NG108-15 cells were exposed to oxidative and inflammatory stress to evaluate the neuroprotective effects of rbMSCs and rcMSCs, as previously described^7^. H_2_O_2_ (Santoku Chemical Industries, Tokyo, Japan) was used to mimic oxidative stimulus, and lipopolysaccharides (LPS) (Wako Pure Chemical Industries) were used to mimic inflammatory stimulus^21,32,52,53^. NG108-15 cells were seeded on culture dishes (Sumitomo Bakelite Co.), and the medium was changed to rbMSC-CM, rcMSC-CM, and control-CM (with 500 mM H_2_O_2_ or 200 ng/mL LPS). Stressed cells were divided into three groups (rbMSC-CM, rcMSC-CM, and control groups) based on the CM that they received. The cells were collected 24 h after exposure to stress. The remaining cells were collected to evaluate the survival rate and mRNA expression of NG108-15 cells.

### Analysis of mRNA expression in stress-exposed NG108-15 cells

Real-time PCR was performed using oligonucleotide primer sets corresponding to the cDNA sequences of rat *Bcl2*, *Bax,* and *Casp3*. In addition, for NG108-15 cells exposed to inflammatory stresses, real-time PCR was performed using oligonucleotide primer sets corresponding to the cDNA sequences of *Tnfrs1a*, *Tlr4*, and *Mlkl*. *Gapdh* was used as an internal control. TaqMan gene primers for expression assays used in this study are listed in Supplementary Table S3 online.

### Statistical analysis

All data are expressed as the mean ± standard deviation of individual samples per group. Statistical analyses were performed using the JMP software from SAS (version 15; SAS Institute Inc., NC, USA). mRNA expressions in rbMSCs and rcMSCs, spinal cord tissue, and in stress-exposed NG108-15 cells were determined using the Mann–Whitney U test. The cavity ratio or survival rate of NG108-15 cells were compared using one-way analysis of variance (ANOVA) with post-hoc Tukey’s honestly significant difference (HSD) test. Two-way ANOVA with post-hoc Tukey’s HSD test was used for analyzing motor function and recovery rate amplitude. *P* < 0.05 was considered significant.

## Supplementary Information

Supplementary Information.

## Data Availability

All data supporting the findings of this study are provided within the manuscript.
