# Supplementary material for: Transplantation of rat cranial bone-derived mesenchymal stem cells promotes functional recovery in rats with spinal cord injury
Source: Sci Rep. 2021 Nov 9;11:21907. doi: 10.1038/s41598-021-01490-1 (PMC8578570; doi:10.1038/s41598-021-01490-1)
Supplement: Supplementary file 1 — Supplementary Information. [file 41598_2021_1490_MOESM1_ESM.docx]

*Supplementary Information*

**Transplantation of Rat Cranial Bone-Derived Mesenchymal Stem Cells Promotes Functional Recovery in Rats with Spinal Cord Injury**

Yuyo Maeda, Takashi Otsuka, Masaaki Takeda, Takahito Okazaki, Kiyoharu Shimizu, Masashi Kuwabara, Masahiro Hosogai, Louis Yuge, and Takafumi Mitsuhara

**Contents:**

**Supplementary Table S1. List of TaqMan assay primers used for real-time PCR involving each type of MSC**

| **Gene** | **Encoded protein** | **Primer sequences (5′–3′)**  **(F: forward, R: reverse)** |
| --- | --- | --- |
| *Bdnf* | Brain-derived neurotrophic factor | F: AGCGCGAATGTGTTAGTGGT  R: GCAATTGTTTGCCTCTTTTTCT |
| *Gapdh* | Glyceraldehyde-3-phosphate dehydrogenase | F: TGGGAAGCTGGTCATCAAC R: GCATCACCCCATTTGATGTT |
| *Gdnf* | Glial cell-derived neurotrophic factor | F: CTAAGATGAAGTTATGGGATGTCG  R: CTTCGAGAAGCCTCTTACCG |
| *Ngf* | Nerve growth factor | F: CAGAGTTTTGGCCTGTGGTC  R: GGACATTACGCTATGCACCTC |
| *Tgfb* | Transforming growth factor-beta | F: CTGGGCACCATCCATGAC R: CAGTTCTTCTCTGTGGAGCTGA |
| *Tsg6* | Tumor necrosis factor-stimulated gene-6 | F: GAAGCAGCCAGAAAGATTGG R: CAACTCTACCCTTGGCCATC |
| *Vegf* | Vascular endothelial growth factor | F: AAAAACGAAAGCGCAAGAAA  R: TTTCTCCGCTCTGAACAAGG |

**Supplementary Table S2. List of TaqMan assays used for real-time PCR involving the spinal cord tissue**

| **Gene symbol** | **Encoded protein** | **Assay ID** |
| --- | --- | --- |
| *Bax* | Bcl2-associated X protein | Rn02532082_g1 |
| *Bcl2* | B-cell leukemia/lymphoma 2 protein | Rn99999125_m1 |
| *Casp3* | Caspase-3 | Rn00563902_m1 |
| *Actb* | Actin beta | Rn00667869_m1 |
| *Il1b* | Interleukin-1 beta | Rn00580432_m1 |
| *Tnfa* | Tumor necrosis factor alpha | Rn99999017_m1 |

**Supplementary Table S3. List of TaqMan assay primers used for real-time PCR involving stress-exposed NG108-15 cells**

| **Gene** | **Encoded protein** | **Primer sequences (5′–3′)**  **(F: forward, R: reverse)** |
| --- | --- | --- |
| *Bax* | Bcl2-associated X protein | F: CCATCATGGGCTGGACAC  R: GCCACCCTGGTCTTGGAT |
| *Bcl2* | B-cell leukemia/lymphoma 2 protein | F: GTACCTGAACCGGCATCTG  R: GGGGCCATATAGTTCCACAA |
| *Casp3* | Caspase-3 | F: CCGACTTCCTGTATGCTTACTCTA  R: CATGACCCGTCCCTTGAA |
| *Gapdh* | Glyceraldehyde-3-phosphate dehydrogenase | F: TGGGAAGCTGGTCATCAAC R: GCATCACCCCATTTGATGTT |
| *Mlkl* | Mixed lineage kinase domain-like | F: TCACTCAGAGACTTAGCCAACG  R: GATGATCTGTCCCAATTTATCCA |
| *Tlr4* | Toll-like receptor 4 | F: CAGGATGATGCCTCTCTTGC  R: TGATCCATGCATTGGTAGGTAA |
| *Tnfrs1a* | Tumor necrosis factor receptor superfamily member 1A | F: CCCAGGGTCTACTCCATCATT  R: CTTCACCCTCCACCTCTTTG |

**Supplementary Table S4. Surface marker expression in MSCs from various tissues**

| **MSC positive markers (%)** | **rbMSCs** | **rcMSCs** |
| --- | --- | --- |
| CD29 | 98.70 ± 1.02 | 99.83 ±0.17 |
| CD44 | 99.30 ± 0.85 | 99.93 ± 0.05 |
| CD90 | 95.87 ± 1.90 | 96.00 ± 3.19 |
| **MSC negative markers (%)** |  |  |
| CD34 | 1.70 ± 0.82 | 0.75 ± 0.45 |
| CD45 | 1.36 ± 1.42 | 0.11 ± 0.11 |

The data represent the mean ± SD of independent experiments (n = 4).

Abbreviations: rbMSCs, bone marrow-derived MSCs; rcMSCs, cranial bone-derived MSCs; SD, standard deviation.


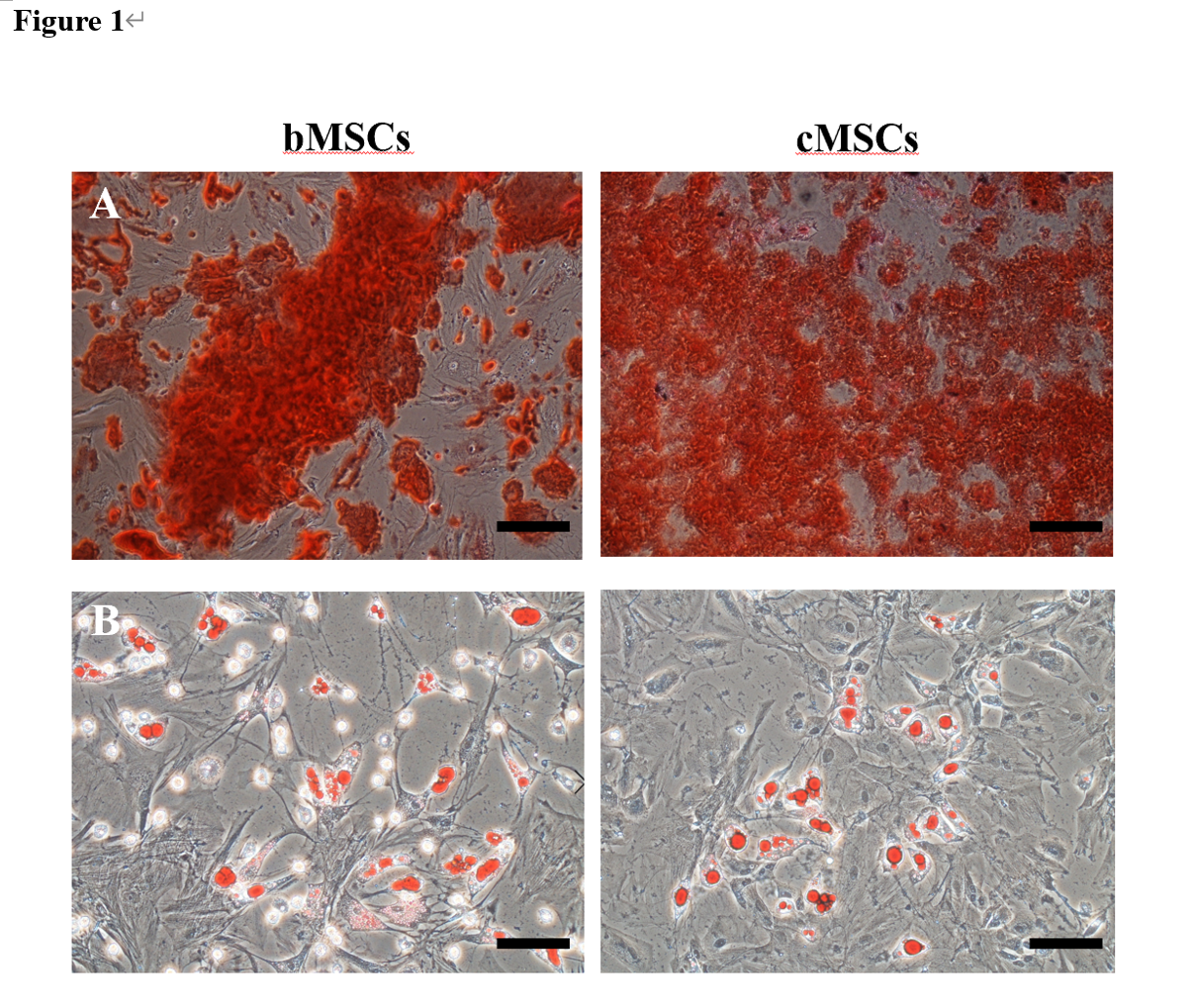


**Supplementary Figure S1.** **Lineage-specific assessment following the differentiation of rbMSC- and rcMSC-derived mesenchymal stem cells.** (A) Alizarin red S staining following the osteogenic differentiation of rbMSCs and rcMSCs. (B) Oil red O staining following the adipogenic differentiation of each MSC. Scale bars, 50 µm.
